# Health action process approach: promoting physical activity, and fruit and vegetable intake among Australian adults

**DOI:** 10.1093/heapro/daad095

**Published:** 2023-08-30

**Authors:** Joy Parkinson, Thomas Hannan, Nicole McDonald, Stephanie Moriarty, Tuyet-Mai Nguyen, Kyra Hamilton

**Affiliations:** Faculty of Law and Business, Australian Catholic University, Banyo, Australia; Griffith Business School, Griffith University, Nathan, Australia; Griffith Business School, Griffith University, Nathan, Australia; Menzies Health Institute of Queensland, Griffith University, Nathan, Australia; Griffith Business School, Griffith University, Nathan, Australia; Griffith Business School, Griffith University, Nathan, Australia; Menzies Health Institute of Queensland, Griffith University, Nathan, Australia; School of Applied Psychology, Griffith University, Mt Gravatt, Australia

**Keywords:** behaviour change, health promotion, health action process approach, non-communicable disease

## Abstract

Based on the health action process approach (HAPA) this study examined whether changes in social cognition constructs could predict change in physical activity and fruit and vegetable intake for adult participants in My health for life, an Australian health promotion behaviour change program. Variance-based structural equation modelling was used to analyse data obtained from Australian adult program participants (*n* = 167) at baseline (T1), week 14 (T2), week 26 (T2), and 6-month post-program (T4). Change scores were calculated for the social cognition constructs and behaviour. Changes in action self-efficacy and outcome expectancies positively predicted changes in intentions. Action self-efficacy changes also predicted changes in maintenance self-efficacy which, in turn, mediated the effect of action self-efficacy on recovery self-efficacy and planning. Planning was predicted by changes in intentions and maintenance self-efficacy. Findings support the use of the HAPA model in designing complex health behaviour change interventions to achieve sustained behaviour change.

**Contribution to Health Promotion**

- Behaviour change programs are argued to be more effective when guided by a theory.
- Findings show applying a theory such as the health action process approach (HAPA) assists to identify levers to build health promotion interventions around leading to the desired behaviour change.
- The My health for life program helped participants increase their self-confidence and planning for undertaking and maintaining physical activity, and fruit and vegetable intake behaviours.
- Including planning for potential setbacks in performing desired behaviours beyond the life of the program is important for long term behaviour maintenance.

## INTRODUCTION

The rise of non-communicable diseases (NCDs), such as heart disease, diabetes, cancer, and obesity, has become a global public health concern (WHO, 2020). These diseases are often linked to unhealthy behaviours such as tobacco use, physical inactivity, and unhealthy diets (AIHW, 2020). Addressing these behaviours through large-scale health promotion programs is an effective strategy to prevent the development and progression of NCDs. Through the promotion of healthy behaviours such as physical activity, fruit and vegetable intake and the creation of supportive environments, large-scale health promotion behaviour change programs can make a significant impact on preventing NCDs and improving overall population health (Michie *et al.*, 2018).

Multi-sectorial, community centred, and evidence-informed approaches are increasingly advocated for to combat the rise in chronic disease (Michie *et al.*, 2018; Fynn *et al.*, 2020). However, they are typically complex due to the various intervention components and the multitude of factors influencing health behaviours (Payne and Thompson, 2015). Consequently, the effectiveness of large-scale initiatives often varies (Dombrowski *et al.*, 2010) and sustaining a newly adopted health behaviour is often difficult for most people (Hills *et al.*, 2014). Therefore, identifying the processes and mechanisms underpinning behaviour change is important to ensure newly adopted health behaviours can be maintained into the future (Michie, 2008).

To improve our understanding of what makes an effective behaviour change intervention, the integration of theoretical models into the design of such programs has become a focus of much behaviour change research (for a comprehensive review, see Hagger *et al.*, 2020). This focus stems largely from arguments behaviour change interventions will be most effective at creating sustained behaviour change if they are based on theoretical models outlined in the behavioural sciences (Prestwich *et al.*, 2014). Theoretical models provide a basis to target key mechanisms in the behaviour change process to increase an individual’s motivation to change or help translate that motivation into actual and sustained behaviour (French *et al.*, 2012; Michie *et al.*, 2014). Identifying the modifiable psychological factors underpinning health behaviours can ultimately contribute to the design of more targeted and effective behaviour change interventions (Michie and Johnston, 2012).

A commonly used theory in health behaviour research is the health action process approach (HAPA; Schwarzer, 2008). The HAPA distinguishes between two phases in the behaviour change process: a motivational phase and a volitional phase (Schwarzer and Hamilton, 2020). Interventions based on the HAPA have demonstrated effectiveness in modifying health behaviours (Zhang *et al.*, 2019; Ahorsu *et al.*, 2020; Scheemann *et al.*, 2020; Asgari *et al.*, 2021; Wu *et al.*, 2022). Such interventions may include behaviour change techniques specifically designed to increase an individual’s motivation to change (i.e. intentions), facilitate the translation of intentions into action (e.g. via planning), or both. For instance, Miller *et al.* (Miller *et al*., 2016) implemented a HAPA-based health promotion behavioural intervention in a sample of prediabetic individuals resulting in a significant improvement in dietary behaviours post-intervention for those in the intervention group, as well as more positive beliefs (i.e. planning, and self-efficacy) regarding diabetes prevention strategies. Furthermore, the extant literature supports the utility of the HAPA constructs in explaining and predicting health behaviour. For example, motivational beliefs (e.g. self-efficacy, intention) and volitional beliefs (e.g. planning, action control) have shown to uniquely predict various health behaviours including dietary behaviours [e.g. (Ochsner *et al.*, 2013)], physical activity (Teleki *et al.*, 2022), oral health hygiene behaviours (Hamilton *et al.*, 2018; Smith *et al.*, 2021; Wu *et al.*, 2022), hand hygiene practices (Reyes Fernández et al., 2015), and social distancing (Hamilton *et al.*, 2020). Thus, the research question for this study is: Can HAPA be used to predict behaviour change in a large health promotion program?

**Fig. 1:**
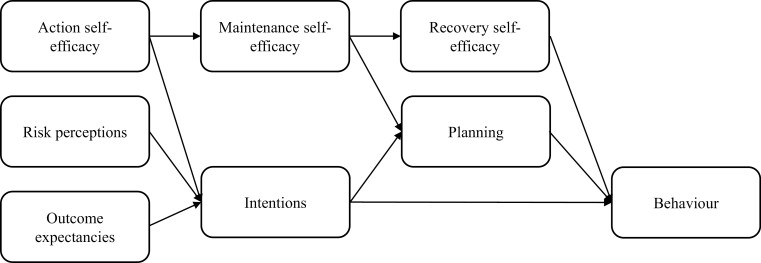
HAPA model and constructs.

The *My health for life* program reported in this study is a government-funded preventive health behaviour change program underpinned by HAPA and implemented in Australia (My health for life, 2019; Parkinson *et al.*, 2022). Designed using an evidence-based co-design and pilot development process (Parkinson *et al.*, 2023) and underpinned by the HAPA model (Schwarzer, 2008), the program comprises six sessions based on the HAPA model delivered by trained facilitators over a 6-month period, followed by an optional post-intervention maintenance program completed online over 6 months (Parkinson *et al.*, 2022; Seib *et al.*, 2022). Over the course of the program, Australian adult participants who are at risk of developing NCDs and deemed eligible for the program, are provided with evidence-based information and activities on how they can modify their health behaviour. Participants set their own Specific, Measurable, Achievable, Realistic, and Timely (SMART) health behaviour goals and are provided with tools and strategies to adopt, achieve, and maintain behaviour change. The program was available free of charge to individuals at risk of developing NCDs. The current study is a smaller study within the overarching program (Parkinson *et al.*, 2022; Seib *et al.*, 2022), examining the impact of the program on social cognitions and behaviour change with a sub-sample (*n* = 167). Specifically, whether the 6-month behaviour change program modified the HAPA social cognition variables and whether these changes successfully predicted physical activity, and fruit and vegetable intake 6-month post-program. The key components of the HAPA addressed in the program as shown in Figure 1 include action self-efficacy, risk perceptions, outcome expectancies, maintenance self-efficacy, recovery self-efficacy, intentions, planning, and their relationship with the target behaviours of fruit and vegetable intake, and physical activity.

## METHODS

### Study design

Quantitative self-report surveys were used to collect demographic information and to measure program participant HAPA constructs and behaviours. Data, collected at four time points, baseline at week 1 (T1), week 12 (T2), week 24 (T3), and at 6-month post-program (T4) via pen and paper surveys were entered into a database by program staff. Ethical clearance was provided by Griffith University Human Research Ethics Committee. All participants provided informed consent to participate in the program.

### Measures

The HAPA constructs were measured with psychometric instruments developed using published guidelines (Schwarzer, 2008) and were adapted for use in the program as shown in Table 1.

**Table 1:** Measures and items

Measure	Details
Risk perceptions	Following the stem ‘If I keep living as I have this far, then I am likely to develop…’, participants rated their perceived risk of developing three chronic health conditions (i.e. type 2 diabetes, heart disease, stroke) Items were rated on a scale ranging from 1 (strongly disagree) to 7 (strongly agree) Scores were averaged with higher scores indicating greater risk perceptions
Outcome expectancies	Following the stem ‘If I do my goal on a regular basis then…’, participants rated three statements I will feel well-balanced and satisfied, I will do something good for my health, *I will feel better afterwards* Items were rated on a scale ranging from 1 (strongly disagree) to 5 (strongly agree) Scores were averaged with higher scores indicating more positive outcome expectancies
Action self-efficacy	I am confident that I can [do my goal] even if it is difficult for me I am certain I can [do my goal] even if it is difficult for me). Items were rated on a scale ranging from 1 (strongly disagree) to 5 (strongly agree) with higher average scores indicating higher action self-efficacy
Recovery self-efficacy	Following the stem ‘I am confident that I can resume…’, participants rated two statements: My goal even if I have interrupted my routine more than once My goal even if I haven’t for several days or weeks Items were rated on a scale ranging from 1 (strongly disagree) to 5 (strongly agree). Higher average scores indicated higher recovery self-efficacy
Maintenance self-efficacy	Following the stem ‘I am confident that I can continuously…’, participants rated three statements [do my goal] on a regular basis even if I have to overcome barriers [do my goal] even if it takes time until it becomes routine [do my goal] on a regular basis even if I need several tries until I am successful Items were rated on a scale ranging from 1 (strongly disagree) to 5 (strongly agree) Higher averaged scores indicated higher maintenance self-efficacy
Planning	Following the stem ‘I have made a detailed plan for my goal so that I can keep going and know in the future…’, participants rated three items What to do if something interferes with my plans How to cope with possible setbacks What to do in difficult situations in order to act according to my intentions Items were rated on a scale ranging from 1 (strongly disagree) to 7 (strongly agree) Higher average scores indicated stronger planning
Intentions	I am planning to [do my goal] on a regular basis I intend to [do my goal] on a regular basis Items were rated on a scale ranging from 1 (strongly disagree) to 5 (strongly agree) Scores were averaged with higher scores indicating stronger intentions
Fruit and vegetable intake	Participants indicated how many serves of fruit and how many serves of vegetables they usually consume on a daily basis. Using national guidelines (National Health and Medical Research Council, 2013) A dichotomous variable was created with participants categorized 0 = insufficient FandV (i.e. less than 5 serves of vegetables and 2 serves of fruit) or 1 = sufficient FandV (i.e. more than 5 serves of vegetables and 2 serves of fruit)
Physical activity	Self-reported physical activity was measured using items from the Active Australia Survey (AIHW, 2003) that asked participants to report on how many times in the previous week they participated in bouts of brisk walking, moderate physical activity, and vigorous physical activity, and for how long (in min) each bout of activity lasted. Using national guidelines for physical activity (AIHW, 2003), total time spent in physical activity was then used to create a dichotomous measure with participants categorized 0 = insufficient physical activity (i.e. less than 150 min of physical activity) or 1 = sufficient physical activity (i.e. more than 150 min of physical activity)

Consistent with the HAPA model (Schwarzer and Hamilton, 2020), we modelled residualized change in action self-efficacy, risk perception, and outcome expectancies to predict changes in intentions. In addition, changes in maintenance self-efficacy were modelled to predicted changes in recovery self-efficacy and mediate the effect of changes in action self-efficacy on changes in recovery self-efficacy. Planning, measured at T3, was expected to be predicted by changes in intentions and maintenance self-efficacy, and mediate the relationship between changes in intention and T4 behaviour. Changes in intention, recovery self-efficacy, and T3 planning were predicted to have direct and positive effects on T4 behaviour. As the HAPA constructs were only measured in T2 and T3 of the program, change in the HAPA constructs was measured as residualized change scores calculated using T2 and T3 data, except for risk perceptions, which included data collected at baseline (T1).

### Participants

Data from 167 program participants (female, 74.3%), collected between February 2017 and November 2019 were used. Participants were included in the analyses if they had completed the full program and attended all four program sessions at which HAPA and behavioural data were collected (i.e. baseline, T1; session 5, T2; session 6, T3; 6-month follow-up, T4). A small number of participants were missing behavioural data at one or more time points for fruit and vegetable intake (*n* = 4) and were subsequently excluded from analyses for that behaviour. Participants provided information on several demographic questions at T1 including sex, education, employment, income, ethnicity, household structure, and postcode. Drawing on postcode data, the Index of Relative Socio-Economic Advantage and Disadvantage (IRSAD; ABS, 2013) was used to provide an indication of participants’ relative socio-economic advantage or disadvantage, with scores ranging from quintile 1 (greatest socio-economic disadvantage) to quintile 5 (greatest socio-economic advantage). Details of participant characteristics are displayed in Table 2.

**Table 2:** Baseline participant characteristics

Characteristic (*n* = 167)	*n* (%)
Gender	
Females	124 (74.3%)
Males	43 (25.7%)
Age	
18–45 years	21 (12.6%)
45+ years	146 (87.4%)
Culturally and linguistically diverse (CALD)	
Yes	8 (4.8%)
No	159 (95.2%)
Aboriginal and Torres Strait Islander	
Yes	5 (3%)
No	162 (97%)
Index of Relative Socio-economic Advantage and Disadvantage (IRSAD)	
Quintile 1	22 (13.2%)
Quintile 2	43 (25.7%)
Quintile 3	19 (11.4%)
Quintile 4	21 (12.6%)
Quintile 5	62 (37.1%)
Education	
Primary education	3 (1.8%)
Secondary education	47 (28.1%)
Certificate or diploma level	59 (35.3%)
Bachelor or postgraduate	53 (31.7%)
Other	3 (1.8%)
Missing	2 (1.2%)
Employment	
Employed full-time	67 (40.1%)
Employed part-time	24 (14.4%)
Home duties	3 (1.8%)
Unemployed	6 (3.6%)
Retired	50 (29.9%)
Permanently ill or unable to work	8 (4.8%)
Other	8 (4.8%)
Missing	1 (0.6%)
Household structure	
Living alone	29 (17.4%)
Living with friends	52 (31.1%)
Living with children	61 (36.5%)
Living with spouse and children	9 (5.4%)
Living with spouse only	6 (3.6%)
Other	9 (5.4%)
Missing	1 (0.6%)
Income	
<$20 000	13 (7.8%)
$20 000 to $39 999	24 (14.4%)
$40 000 to $59 999	24 (14.4%)
$60 000 to 79 999	14 (8.4%)
$80 000 to $99 999	14 (8.4%)
More than $100 000	40 (24%)
Missing	39 (23.4%)

### Statistical analyses

Descriptive data were analysed using SPSS version 23. Descriptive data are expressed as counts and percentages, mean, standard deviation (SD), and bivariate statistics were performed using chi-square tests and ANOVA with statistical significance set at *a* = 0.05. Missing data analysis for the HAPA constructs revealed 4.34% of the data were missing, *χ*^2^ (1030) = 1134.56, *p* = 0.012. Further inspection of the missing data revealed no clear item-level pattern in the missingness, suggesting the data were missing at random (MAR). The validity of this assumption was examined by inspecting the missing-data patterns and modelling the missingness of the data against other available explanatory variables (Enders, 2010). Assuming the data were MAR, an expectation maximization (EM) algorithm was therefore used to impute the missing item-level data for the HAPA constructs (Graham, 2009).

Residualized change scores were computed by regressing the final measurement (T4) onto previous and baseline measurements. In this way, the residualized score is a post-test score with the previous measurements partialed out and represents the amount of change in a construct given baseline assessment (Castro-Schilo and Grimm, 2018). Residualized scores eliminate autocorrelated error and regression to the mean effects and are therefore preferable than simple change scores (Castro-Schilo and Grimm, 2018). As the HAPA constructs were not measured at baseline, residualized change scores for these variables were computed by regressing variables at T3 (session 6) onto the same variables at T2 (session 5), except for risk perceptions which included data from baseline, T2, and T3.

For structural equation models, a confirmatory factor analysis (CFA) was first performed in AMOS (version 27) to assess the reliability and validity of the HAPA measures. The hypothesized structural models were then tested using variance-based structural equation modelling (VB-SEM) using the statistical software Warp PLS v.7.0 (Kock, 2015). VB-SEM analysis uses latent variables to explicitly model measurement error and, thus, is similar to covariance-based SEM analyses. Compared to covariance-based SEM analyses, however, the partial least squares algorithm used in VB-SEM is based on ranked data. As a result, VB-SEM is distribution free which means the estimation is subsequently less affected by factors such as model complexity, the presence of non-normality in the data, and small sample size (Kock, 2015).

Goodness-of-fit (GoF) indices assessed the adequacy of the estimated model. Specifically, the overall GoF index with values of 0.10, 0.25, and 0.36 used to indicate small, medium, and large effects, respectively (Tenenhaus *et al.*, 2005), average full-variance inflation factor (AVIF), which is recommended to be less than 5.00 (ideally less than 3.30) for a well-fitting model (Koch, 2015), the average path coefficient (APC), and average *R*^2^ (ARS) coefficient. A bootstrap resampling method with 100 replications was used to test the hypothesized direct and indirect effects (Kock, 2015). Separate models were run for prediction of fruit and vegetable intake, and physical activity. In addition, age category (i.e. <45 or 45+ as prescribed by program eligibility criteria), gender, mode of program delivery, and Index of Relative Socio-economic Advantage and Disadvantage (IRSAD) scores were entered into the models as covariates alongside measures of behaviour at baseline, T2, and T3.

## RESULTS

### Descriptives

Table 3 displays descriptives and results of paired samples *t*-test for the HAPA constructs across T2 and T3. Only risk perception was found to significantly decrease from T2 to T3. Correlations between residualized change in HAPA constructs and behaviour are shown in Supplementary material.

**Table 3:** Means, standard deviations, and difference test of the health action process approach constructs at each time point

Variable	T2 (session 5)	T3 (session 6)	*t*	95% CI	
	*M* (SD)	*M* (SD)		LL	UL
Risk perceptions	4.11 (1.86)	3.55 (1.78)	3.95***	0.28	0.84
Action self-efficacy	3.98 (0.87)	4.07 (0.84)	−1.32	−0.23	0.05
Outcome expectancies	4.78 (0.57)	4.75 (0.45)	0.54	−0.08	0.14
Intentions	4.49 (0.69)	4.52 (0.69)	−0.49	−0.14	0.09
Maintenance self-efficacy	4.23 (0.68)	4.18 (0.80)	0.80	−0.07	0.18
Recovery self-efficacy	4.26 (0.80)	4.21 (0.90)	0.73	−0.09	0.19
Planning	–	5.37 (1.23)			

CI, confidence interval; LL, lower limit; UL, upper limit.

***p < 0.001.

### Confirmatory factor analysis

Confirmatory factor analysis assessed the latent HAPA constructs for scale reliability and validity. As seen in Table 4, all standardized factor loadings for the latent variables exceeded 0.80 and were statistically significant (*p* < 0.05). In addition, the coefficients of standardized loadings all exceeded 0.5 and their average loadings were found to be greater than 0.7, indicating good internal consistency (Hair *et al*., 2010). Further, convergent validity was demonstrated for the measures as evidenced by AVE values exceeding 0.50 and factor composite reliability values were all above the recommended value of 0.60 (Fornell and Larcker, 1981). In addition, discriminant validity, demonstrated by the square root of each construct, AVE found to exceed the value of all between-construct correlations (Barclay *et al*., 1995). As shown in Table 4, the overall fit statistics demonstrated a good fit of the measurement model to the data (Bentler, 1990, 2007).

**Table 4:** Factor loadings and reliability indices for the HAPA items

Factor loadings and reliability	Loads (beta)	SE	C.R	Average load	AVE	Composite reliability
*Action self-efficacy*				0.908	0.823	0.903
Goal confident if difficult.	0.917	0.059	16.875			
Goal certain if difficult.	0.898	0.059	16.875			
*Risk perceptions*				0.929	0.866	0.951
Knowledge lifestyle type.	0.847	0.047	16.902			
Knowledge lifestyle heart disease.	0.983	0.075	16.902			
Knowledge lifestyle stroke.	0.956	0.074	15.87			
*Outcome expectations*				0.908	0.825	0.933
Goal balanced satisfied.	0.889	0.051	23.079			
Goal good for health.	0.939	0.037	23.079			
Goal feel better.	0.896	0.039	24.576			
*Intentions*				0.913	0.833	0.909
Goal planning regular basis.	0.911	0.059	15.66			
Goal intend regular basis.	0.914	0.069	15.66			
*Maintenance self-efficacy*				0.932	0.870	0.952
Goal continuously several tries.	0.898	0.076	14.044			
Goal continuously routine.	0.917	0.067	14.044			
Goal continuously overcome barriers.	0.892	0.059	18.544			
*Recovery self-efficacy*				0.958	0.918	0.957
Goal confident interrupted.	0.957	0.061	17.004			
Goal confident lapse.	0.932	0.056	17.004			
*Planning*				0.902	0.814	0.929
Goal interfere plan.	0.908	0.047	20.048			
Goal cope setbacks.	0.976	0.052	20.048			
Goal difficult situations.	0.940	0.054	18.367			
Goodness-of-fit	RMSEA	CFI	TLI	NFI		
*χ* ^2^ = 188.511, df = 125, *χ*^2^/df = 1.508 (*p* = 0.000)	0.055	0.978	0.974	0.939		

### Structural equation models: model fit indices

The model predicting change in fruit and vegetable intake demonstrated good fit to the data (GoF = 0.560; ARS = 0.314, *p* < 0.001; APC = 0.238, *p* < 0.001; AVIF = 1.158) and explained 16% of the variance in behaviour change. The model predicting physical activity also demonstrated good fit to the data (GoF = 0.564; ARS = 0.318, *p* < 0.001; APC = 0.205, *p* = 0.002; AVIF = 1.586) and explained 18.2% of the variance in behaviour. The direct and indirect effects, including effect sizes, for the models predicting fruit and vegetable intake and physical activity are displayed in Table 5.

**Table 5:** Standardized path coefficients and effect sizes for the direct and indirect effects

Path	*β*	*p*	*ƒ* ^2^
Direct effects fruit and vegetable intake			
Action self-efficacy → Maintenance self-efficacy	0.597	**<0.001**	0.356
Action self-efficacy → Intentions	0.469	**<0.001**	0.239
Outcomes expectancies → Intentions	0.138	**0.036**	0.032
Risk Perceptions → Intentions	−0.069	0.185	0.011
Maintenance self-efficacy → Recovery self-efficacy	0.734	**<0.001**	0.539
Maintenance self-efficacy → Planning	0.359	**<0.001**	0.162
Intentions → Planning	0.188	**0.007**	0.069
Intentions → Behaviour	0.023	0.385	0.003
Planning → Behaviour	0.283	**<0.001**	0.092
Recovery self-efficacy → Behaviour	0.159	**0.019**	0.039
Mode of delivery → Behaviour	0.072	0.177	0.003
Gender → Behaviour	0.051	0.255	0.003
IRSAD → Behaviour	0.141	**0.033**	0.021
Age → Behaviour	−0.053	0.248	0.003
Indirect effects fruit and vegetable intake			
Action self-efficacy → Maintenance self-efficacy → Recovery self-efficacy	0.438	**<0.001**	0.221
Action self-efficacy → Maintenance self-efficacy → Planning	0.215	**<0.001**	0.116
Action self-efficacy → Intentions → Planning	0.088	0.053	0.048
Action self-efficacy → Intentions → Behaviour	0.011	0.424	0.002
Outcome expectancies → Intentions → Planning	0.026	0.319	0.007
Outcome expectancies → Intentions → Behaviour	0.003	0.477	0.000
Risk Perceptions → Intentions → Planning	−0.013	0.407	0.002
Risk Perceptions → Intentions → Behaviour	−0.002	0.489	0.000
Intentions → Planning → Behaviour	0.053	0.166	0.002
Maintenance self-efficacy → Planning → Behaviour	0.102	**0.031**	0.022
Maintenance self-efficacy → Recovery self-efficacy → Behaviour	0.117	**0.016**	0.025
Direct effects physical activity			
Action self-efficacy → Maintenance self-efficacy	0.602	**<0.001**	0.362
Action self-efficacy → Intentions	0.468	**<0.001**	0.239
Outcome expectancies → Intentions	0.140	**0.032**	0.033
Risk Perceptions → Intentions	−0.070	0.181	0.012
Maintenance self-efficacy → Recovery self-efficacy	0.732	**<0.001**	0.535
Maintenance self-efficacy → Planning	0.350	**<0.001**	0.156
Intentions → Planning	0.194	**0.005**	0.071
Intentions → Behaviour	0.061	0.211	0.005
Planning → Behaviour	0.139	**0.033**	0.013
Recovery self-efficacy → Behaviour	−0.097	0.101	0.011
Mode of delivery → Behaviour	0.109	0.076	0.014
Gender → Behaviour	0.058	0.225	0.005
IRSAD → Behaviour	0.166	**0.014**	0.030
Age → Behaviour	−0.083	0.137	0.004
Indirect effects physical activity			
Action self-efficacy → Maintenance self-efficacy → Recovery self-efficacy	0.440	**<0.001**	0.221
Action self-efficacy → Maintenance self-efficacy → Planning	0.211	**<0.001**	0.112
Action self-efficacy → Intentions → Planning	0.091	**0.046**	0.048
Action self-efficacy → Intentions → Behaviour	0.029	0.299	0.002
Outcome expectancies → Intentions → Planning	0.027	0.309	0.007
Outcome expectancies → Intentions → Behaviour	0.009	0.438	0.001
Risk perceptions → Intentions → Planning	−0.014	0.402	0.003
Risk perceptions → Intentions → Behaviour	−0.004	0.469	0.001
Intentions → Planning → Behaviour	0.051	0.175	0.004
Maintenance self-efficacy → Planning → Behaviour	0.049	0.185	0.001
Maintenance self-efficacy → Recovery self-efficacy → Behaviour	−0.071	0.095	0.002

Bold p values represent significance at < .05 level.

### Fruit and vegetable intake

#### Direct effects

Results as shown in Table 5 revealed a positive direct effect of changes in action self-efficacy (*β* = 0.469, *p* < 0.001) and outcome expectancies (*β* = 0.138, *p* = 0.036) on changes in intentions; however, there was no direct effect of changes in risk perceptions on intentions (*β* = −0.069, *p* = 0.185). There was also a significant direct effect of changes in action self-efficacy on changes in maintenance self-efficacy (*β* = 0.597, *p* < 0.001), as well as significant direct effects of changes in maintenance self-efficacy on changes in recovery self-efficacy (*β* = 0.734, *p* < 0.001) and planning (*β* = 0.359, *p* < 0.001). In addition, a significant direct effect was found between changes in intentions and planning (*β* = 0.188, *p* = 0.007). In the prediction of behaviour change, no significant direct effect was observed for changes in intentions (*β* = 0.023, *p* = 0.385), however, a significant positive effect was found for planning (*β* = 0.283, *p* ≤ 0.001) and changes in recovery self-efficacy (*β* = 0.159, *p* = 0.019) on changes in behaviour.

#### Indirect effects

Results as shown in Table 5 revealed changes in maintenance self-efficacy significantly mediated the effects of changes in action self-efficacy on both recovery self-efficacy (*β* = 0.438, *p* < 0.001) and planning (*β* = 0.215, *p* < 0.001). Results also revealed the effects of changes in maintenance self-efficacy on changes in behaviour were mediated by changes in recovery self-efficacy (*β* = 0.112, *p* = 0.020) and planning (*β* = 0.128, *p* = 0.010). Planning did not significantly mediate the effects of changes in intentions on changes in behaviour (*β* = 0.053, *p* = 0.166).

### Physical activity

#### Direct effects

For the model predicting physical activity as shown in Table 5, there was a positive direct effect of changes in action self-efficacy (*β* = 0.468, *p* < 0.001) and outcome expectancies (*β* = 0.140, *p* = 0.032) on changes in intentions; yet there was no direct effect of changes in risk perceptions on intentions (*β* = −0.070, *p* = 0.181). Changes in action self-efficacy had a direct effect on changes in maintenance self-efficacy (*β* = 0.602, *p* < 0.001), and changes in maintenance self-efficacy had a direct effect on changes in recovery self-efficacy (*β* = 0.732, *p* < 0.001) and planning (*β* = 0.350, *p* < 0.001). A significant direct effect was also found between changes in intentions and planning (*β* = 0.194, *p* = 0.005). In the prediction of change in physical activity, only planning had a positive direct effect on behaviour (*β* = 0.139, *p* = 0.033). Changes in intentions (*β* = 0.061, *p* = 0.211) and recovery self-efficacy (*β* = −0.097, *p* = 0.101) did not directly predict behaviour.

#### Indirect effects

As shown in Table 5 changes in maintenance self-efficacy significantly mediated the effects of changes in action self-efficacy on both recovery self-efficacy (*β* = 0.440, *p* < 0.001) and planning (*β* = 0.211, *p* < 0.001). Changes in intentions were also found to mediate the relationship between changes in action self-efficacy and planning (*β* = 0.091, *p* = 0.046). Planning did not mediate the effects of changes in intentions on change in physical activity (*β* = 0.051, *p* = 0.175).

## DISCUSSION

The aim of this study was to apply the HAPA model and determine whether changes in social cognitions were predictive of change in physical activity and fruit and vegetable intake for program participants. Changes in both action self-efficacy and positive expected outcomes were found to directly predict change in intention, supporting the theorized motivational phase of the HAPA (Schwarzer, 2008). Consistent with Zhang *et al.* (Zhang *et al.*, 2019) meta-analyses, changes in perceived risk did not significantly predict changes in intentions. Although risk perception is often modelled as a determinant of motivation (Hamilton *et al.*, 2020; Schwarzer and Hamilton, 2020), previous intervention studies have similarly found risk perception to have no direct effect on health-related intentions [e.g. (Hattar *et al.*, 2016; Pinidiyapathirage *et al.*, 2018; Zhang *et al.*, 2019)]. These findings suggest for participants in the program, motivation to change their preventive health behaviours may be more strongly influenced by their belief in their ability to make a healthy behaviour change (i.e. action self-efficacy) and their beliefs that modifying their health behaviours will subsequently result in positive outcomes (i.e. positive outcome expectancies), more so than the perceived risks of not modifying their health behaviours. This highlights the need for interventions to identify key beliefs likely to influence a person’s motivation to modify their behaviour (Michie *et al.*, 2018). Once identified, intervention strategies can specifically target those beliefs and increase a person’s motivation to change [e.g. (Miller *et al.*, 2016)].

Changes in action self-efficacy were found to significantly predict planning via changes in both intentions and maintenance self-efficacy, consistent with prior research (Hatter and Hagger, 2016). This highlights the important role self-efficacy beliefs play in the behaviour change process (Ochsner *et al.*, 2013). Individuals who held strong beliefs in their ability to modify their health behaviours reported having stronger intentions to modify their behaviour and had stronger beliefs in their ability to maintain their newly adopted behaviour. Changes in these beliefs subsequently predicted increased planning that would help them to cope with and manage potential setbacks or adversities. Interventions attempting to increase people’s motivation to modify their physical activity and fruit and vegetable intake and maintain newly adopted behaviours should therefore focus on action self-efficacy strategies (Luszczynska *et al.*, 2007).

Forming plans was identified as playing an important role in post-program behaviour which is consistent with prior research (Kwasnicka *et al.*, 2013). People who formed strong plans were better equipped to act on their behaviour change goals than those with weak plans, and subsequently more likely to modify their behaviours including physical activity and fruit and vegetable intake. This is consistent with previous research where creating concrete plans regarding how to handle and respond to unexpected or adverse events is an important self-regulatory ability (Schwarzer and Hamilton, 2020). This indicates that program managers should focus on strategies that assist participants to create strong plans for achieving their desired behavioural goals. Likewise, recovery self-efficacy was identified as effecting behaviour change and highlights the importance of implementing strategies to increase participants beliefs in their ability to not only modify, but also to successfully regulate their behaviour to achieve their desired outcomes.

Planning appeared to have a stronger role in regulating participants’ behaviour over time, compared to their intentions, by aiding in the automatization of behaviours via the formation of habits (i.e. automatic cue-behaviour associations; Gardner and Rebar, 2019). Planning as a significant predictor of behaviour change, while intentions were not, provides some support to this possibility, as planning has been found to be an important factor in habit formation [e.g. (Potthoff *et al.*, 2017)]. Future research could consider measuring participants’ health-related habits to ascertain whether changes in habits during behaviour change programs can explain additional variance in health behaviour change over and above constructs like intentions and planning [e.g. (Hamilton *et al.*, 2019)].

The current study is not without limitations. First, the current sample consisted predominantly of females and as such, these findings should be replicated using a larger sample comprising of more males to ensure the generalisability of these findings. Second, because the program is a public health program there was no control group used in this study. As a result, no inferences regarding causality can be drawn from these findings. Third, although the time gap between the intervention and the follow-up survey provides some evidence for sustained behaviour change, the measurement of additional constructs not assessed in this study would provide greater insight into the factors contributing to behaviour change. For example, habits are automatic impulses to perform an action in response to associated cues and have been identified as a key construct in behaviour maintenance (Gardner and Rebar, 2019). The use of planning by those in the program may facilitate the development of strong habitual responses to health-related cues (Fleig *et al.*, 2013), which then promotes continued behaviour. Future research could measure habit to identify how specially planning contributes to sustained behaviour for those in the program. This study does not report on HAPA constructs at baseline to not account for previous assessments, as these may compromise the true effects of social-cognitive variables on the target behaviours (Bandura, 1986). This study employed only coping planning, which is a limitation and future studies should also include action planning (Schwarzer, 2008). The program studied was primarily targeted at participants aged over 45 years as per the program’s eligibility criteria, therefore limiting implications for people aged under 45 years of age. Lastly, the current study relied on the use of self-report measures of behaviour which may be influenced by self-report bias (Shim *et al.*, 2014). Utilizing objective measures of behaviour, like pedometers or daily reflective journals [e.g. (Salmon *et al.*, 2016)], may provide a more accurate measure of behaviour and behaviour change.

## CONCLUSION

Taken together, the findings have important implications for intervention design and behaviour change research. Specifically, the current study provides further support for the use of the HAPA model in designing complex health behaviour change interventions. Understanding the social cognition variables which influence health behaviours is important for the development of effective interventions that contribute to sustained behaviour change. The findings suggest targeting the HAPA constructs underpinning participants’ motivation to modify their health behaviours and encouraging them to form plans which help to protect their goals from potential setbacks are effective strategies for promoting positive health behaviour change.

## Supplementary Material

daad095_suppl_Supplementary_Material
