# Supplementary material for: Health action process approach: promoting physical activity, and fruit and vegetable intake among Australian adults
Source: Health Promot Int. 2023 Aug 30;38(4):daad095. doi: 10.1093/heapro/daad095 (PMC10468016; doi:10.1093/heapro/daad095)
Supplement: daad095_suppl_Supplementary_Material [file daad095_suppl_supplementary_material.docx]

| Variable | 1 | 2 | 3 | 4 | 5 | 6 | 7 | 8 | 9 |
| --- | --- | --- | --- | --- | --- | --- | --- | --- | --- |
| 1. Action self-efficacy | - |  |  |  |  |  |  |  |  |
| 2. Risk perceptions | -.18^*^ | - |  |  |  |  |  |  |  |
| 3. Outcome expectancies | .23^**^ | .06 | - |  |  |  |  |  |  |
| 4. Intentions | .50^**^ | -.12 | .23^**^ | - |  |  |  |  |  |
| 5. Recovery self-efficacy | .50^**^ | -.08 | .28^**^ | .55^**^ | - |  |  |  |  |
| 6. Maintenance self-efficacy | .60^**^ | -.12 | .33^**^ | .60^**^ | .72^**^ | - |  |  |  |
| 7. Planning | .53^**^ | -.20^**^ | .26^**^ | .32^**^ | .49^**^ | .41^**^ | - |  |  |
| 8. Fruit and vegetable intake | .23** | -.16* | .14 | .13 | .17* | .21** | .30** | - |  |
| 9. Physical activity | .06 | -.13 | .08 | -.01 | -.08 | -.03 | .12 | .05 | - |

**Supplementary file.** Correlations between Residualized Change in HAPA constructs and behaviour *Notes.* **p*< .05 ***p*<.01, ****p*< .001
